# Plant Growth Biostimulants Based on Different Methods of Seaweed Extraction with Water

**DOI:** 10.1155/2016/5973760

**Published:** 2016-06-06

**Authors:** Katarzyna Godlewska, Izabela Michalak, Łukasz Tuhy, Katarzyna Chojnacka

**Affiliations:** Department of Advanced Material Technologies, Faculty of Chemistry, Wrocław University of Technology, Smoluchowskiego 25, 50-372 Wrocław, Poland

## Abstract

We explored two methods for obtaining aqueous extracts: boiling and soaking of Baltic seaweeds (EB and ES, resp.). Algal extracts were characterized in terms of polyphenols, micro- and macroelements, lipids content, and antibacterial properties. The utilitarian properties were examined in the germination tests on* Lepidium sativum *for three extract dilutions (0.5, 2.5, and 10%). It was found that the extracts were similar in micro- and macroelement concentrations. Water was proved to be a good solvent to extract phenolic compounds. The algal extract produced by soaking biomass did not show inhibitory effect on* Escherichia coli *and* Staphylococcus aureus*. Only the boiled extract had an inhibitory activity against* E. coli*. Germination tests revealed a positive influence of the bioproducts on the cultivated plants. In the group treated with 10% EB, plants were 13% longer than in the control group; the content of elements B, Mo, Zn, and Na in the group treated with 10% ES was higher by 76%, 48%, 31%, and 59% than in the control group, respectively; the content of chlorophyll was 2.5 times higher in 0.5% ES than in the control group. Extracts showed the slight impact on the morphology of plants.

## 1. Introduction

Marine algae are considered to be one of the most important sustainable resources [1] with industrial potential [2]. Composition of macroalgae provides an excellent opportunity to study a diversity of rare biologically active compounds [3, 4] that show an array of physiological and biochemical characteristics [4, 5]. Extracts derived from algae contain such components as polysaccharides (e.g., galactan, fucoidan, alginate, and laminarin), proteins (e.g., lectins), polyunsaturated fatty acids (PUFAs), pigments (e.g., chlorophylls, carotenoids, and phycobiliproteins), polyphenols (e.g., phenolic acids, flavonoids, cinnamic acid, isoflavones, benzoic acid, and lignans, quercetin), minerals (e.g., K, Mg, Ca, and Na), and plant growth hormones (e.g., cytokinins, auxins, gibberellins, and abscisic acid) [6]. Scientific research has proven that some algal metabolites show potential antioxidant, antiproliferative [7], antidiabetic [8], antitumor [9], anti-inflammatory [10], antiallergic [11], and anti-HIV properties [12]. Because of their composition and the functional properties they are used as human food [13, 14], especially in Asia (China, Japan, and Korea) and as animal feed [15–17]. Due to their growth-stimulating activities algal formulations are used as biostimulant in crop production [15, 18, 19]. Seaweed liquid extracts have become more significant in agriculture as foliar sprays because they contain promoting hormones or trace elements (Fe, Cu, Zn, and Mn) which, added to the soil or applied to seeds, stimulate plant growth [15]. Bioproducts used in agriculture and horticulture are mainly prepared from brown seaweeds of* Ascophyllum nodosum, Ecklonia maxima,* and* Macrocystis pyrifera* [20].

The seaweed biomass collected from the Baltic Sea could be the raw material for the production of algal extracts [21]. Extraction is the most important and starting step in isolating different types of components. In the case of seaweed the extraction efficiency is reduced due to the presence of complex cell wall, which could be affected by solvent composition, temperature, time, and pH [22]. Different extraction techniques have been used to maximize biologically active compounds isolation from plant material [23], for example, microwave assisted extraction [24, 25], supercritical fluid extraction with carbon dioxide as a solvent, Soxhlet extraction [26], enzyme-assisted extraction [27], and ultrasound-assisted extraction [28]. To this purpose different solvents can be used like ethanol, acetone, methanol-toluene [29], methanol, petroleum ether, ethyl acetate, dichloromethane, and butanol [30]. These methods require the use of expensive and toxic solvents.

To overcome limitations of conventional extraction methods and to produce algal extract we chose boiling and soaking extraction methods with distilled water. These processes are environment friendly and they do not require organic solvents. The algal extracts that we received were characterized in terms of the following:

 Polyphenols.
 Lipids (n-3 and n-6 fatty acids).
 Microelements (B, Co, Cu, Fe, Mn, Mo, Ni, Si, and Zn).
 Macroelements (Ca, K, Mg, Na, P, and S).
 Antibacterial properties (gram-negative:* Escherichia coli* and gram-positive:* Staphylococcus aureus*).

We conducted germination tests on* Lepidium sativum* to examine the utilitarian properties and we determined the content of nutrients and chlorophyll in the cultivated plants and their morphology (using Scanning Electron Microscopy). The algal extract with a high content of biologically active compounds may find its future application in various industries.

## 2. Materials and Methods

### 2.1. Chemicals

All the reagents used in the experiment were of an analytical grade. Sodium carbonate, ethanol, methanol, and nitric acid were purchased from POCH SA (Poland). Folin-Ciocalteu's phenol reagent, gallic acid, and nitric acid 69% m/m, spectrally pure (Suprapur), were purchased from Merck KGaA (Darmstadt, Germany).

### 2.2. Collection of Algae

The seaweeds (*Polysiphonia, Ulva, *and* Cladophora*) were collected from the Baltic Sea (Sopot, Poland), in August 2013. Algae floating freely in the coastal zone were collected from the water to minimize the contamination of raw biomass. Subsequently, the algal material was rinsed with water in order to purify it from salt and sand. Next the larger impurities (e.g., sea shells and pieces of wood) were separated; then the biomass was dried to 15% of moisture, and finally it was ground to particle size <0.3 mm [31].

### 2.3. Extract Production

We applied two extraction methods according to the modified procedures described by Pise and Sabale [32]. Seaweed extracts were prepared in such a way that we added 50 g of dried and shredded biomass to 150 mL of distilled water (250 mL flask). The solution was boiled in water bath for 30 minutes. In the second method we added 150 mL of distilled water to 50 g of prepared algal biomass and left it for 2 days. Afterwards each sample was centrifuged at 4250 rpm for 5 minutes and filtered with Whatman number 1 filter paper. The supernatant that we acquired was taken as a 100% algal liquid extract. For germination tests algal liquid extracts were prepared with different doses of 0.5, 2.5, and 10%. The effect of the produced extracts on the weight and height as well as chemical composition of* Lepidium sativum* was tested. We marked the extract obtained by boiling extraction as EB and by soaking extraction as ES.

### 2.4. Characteristics of Algal Extract

The methodology was based on the procedures described by Michalak et al. [25].

#### 2.4.1. Multielemental Composition of Algal Extracts

Firstly, the samples of the algal biomass and cultivated plants (0.5 g) were purified from organic matter with nitric acid (5 mL) in Teflon bombs in a microwave oven (Milestone Start D, USA). Secondly, samples were diluted with redemineralized water (Millipore Simplicity) to 50 g. The samples were analyzed in three repetitions (presented as arithmetic mean, the relative standard deviation was <5%). Finally, we determined the content of elements in algal extracts, samples of algal biomass, and cultivated plants by ICP-OES (iCAP 6500 Duo, Thermo Scientific, USA) [25].

#### 2.4.2. Phenolic Compounds in the Algal Extracts

The phenolic compounds concentrations in algal extracts expressed as gallic acid equivalents were determined with the Folin-Ciocalteu reagent [25].

#### 2.4.3. Antibacterial Assay

Antibacterial activity (*Escherichia coli* and* Staphylococcus aureus*) was determined by the Kirby Bauer disk diffusion method, and it was recorded by measuring the diameter of the zone of inhibition (gentamicin was used as a the reference antibiotic) [25].

### 2.5. Utilitarian Properties of Algal Extracts

#### 2.5.1. Germination Tests: Petri Dish Tests

To evaluate useful properties of the algal extracts, we performed the germination tests (three replicates on Petri dishes (8.9 cm), 50 seeds each) with garden cress (*Lepidium sativum*). Experiments were conducted in standardized conditions on the Jacobsen apparatus. Then each dish was treated with appropriate algal extract (5 mL). The control group (C) was treated with distilled water (5 mL). After three days, all dishes were treated with the same subsequent doses of extract/water. The tests were performed for 7 days, after which we weighed the plants and measured the height of shoot length [25].

#### 2.5.2. Chlorophyll Content in the Cultivated Plants

To determine plant pigments, we subjected the aerial parts of cultivated garden cress to a 30-minute methanolic extraction process. The resultant colored solution was analyzed by UV-Vis spectrophotometer (Varian Cary 50 Conc. Instrument, Victoria, Australia). Measurements were made at wavelengths of *λ* = 663 and 645 nm. The concentration of total chlorophyll (Total Chl), chlorophyll* a* (Chl(*a*)), and chlorophyll* b* (Chl(*b*)) was determined from the equations [33]:

(1) Total  Chl=8.02·A663+20.2·A645,Chla=12.7·A663−2.69·A645,Chlb=22.9·A645−4.68·A663.

#### 2.5.3. SEM Analysis of Cultivated Plants

Stalk and leaf (the internal and external part) of* Lepidium sativum* were examined by Scanning Electron Microscopy at Wrocław University of Environmental and Life Sciences (Electron Microscope Laboratory). The samples were examined with a Scanning Electron Microscope-EVOLS 15 Zeiss (Oberkochen, Germany) operating at 20 kV. For the test, SE1 detector was used [25].

### 2.6. Statistical Analysis

The results were elaborated statistically by* Statistica ver. 10* (significantly different when *p* < 0.05). Distribution normality of the experimental results was assessed by the Shapiro-Wilk test whereas group differences were investigated by means of the Tukey test.

## 3. Results and Discussion

### 3.1. Characteristics of Algal Extract

#### 3.1.1. Multielemental Composition of Algal Extracts

Table 1 presents the multielemental composition of raw algal biomass and extracts obtained by boiling and soaking with water. Generally, the extracts were similar in terms of the elemental composition. EB was especially rich in P, S, and B; on the other hand the ES contained a great amount of Ca, Mg, and Fe. It should be noted that toxic elements were extracted from the raw algal biomass in low amounts.

The multielemental composition of algal extracts resulting from different extraction methods has been known for some time (Table 1). Selvam and Sivakumar [34] presented the composition of* Ulva reticulata* extract (obtained by adding 500 g of powdered seaweeds to 5 L of water and boiling for 45 min at 60°C in a plugged conical flask). This extract was richer in ions such as Cu and Zn than extracts presented in this study (EB and ES), but Ca, K, Mg, and Na concentrations were much lower as compared to those of the Baltic extract. The Fe ions concentration (5.22 mg/L) reported in their work was higher than in EB (2.53 mg/L) but lower than in ES (17.6 mg/L). Sivasangari et al. [35] examined the mineral composition of two extracts obtained by boiling the seaweeds* Sargassum wightii *and* Ulva lactuca*. As it was shown, the macroelements content such as K, Mg, and Na was much lower than in that of Baltic extracts, but Fe ions concentration was higher. These extracts contained more microelement ions, for example, Co, Cu, and Zn, than extract EB and ES. The differences may be attributed to the mineral composition of the raw biomass and the extraction methods used. Michalak et al. [25] present the extracts obtained by microwave assisted extraction (MAE) in different temperatures (25, 40, and 60°C). It can be seen that at a lower extraction temperature, there was a lower elements concentration in the final extract. ES contained higher levels of macroelements but simultaneously contained the smallest amount of phosphorus. Microelements in all presented products were extracted at a similar level. ES contained the highest amount of Fe ions.

#### 3.1.2. Polyphenols in Algal Extracts

Polyphenols constitute a heterogeneous group of molecules which provide a wide range of potential biological activity [36]. This class of compounds includes phenolic acids, lignins, flavonoids, tocopherols, and tannins. The use of natural antioxidants and antimicrobials can reduce the application of synthetic forms such as butylated hydroxyanisole (BHA) and butylated hydroxytoluene (BHT) [37, 38]. Scientific research shows that polyphenols are good antioxidants and are effective in preventing cardiovascular and inflammatory diseases and also can be used as cancer chemopreventing agents [39]. In addition to the polyphenols from terrestrial plants (derived from gallic and ellagic acid), seaweeds have been shown as a rich source of different types of polyphenols (derived from phloroglucinol units) with unique structural properties [36, 37, 39–41]. For example,* Halimeda* (*Chlorophyceae*) contains high concentrations of polyphenols such as catechin, epicatechin, epigallocatechin gallate, and gallic acid [40]. The highest contents of these compounds are found in brown seaweeds [36].

We found out that the EB extract contained higher concentrations of polyphenols (215 mg/L) than the ES extract (173 mg/L). Water is regarded as a good solvent for isolation of phenolic compounds. López et al. [41] prepared seaweed extract by mixing (with a magnetic stirrer) dried algal powder (brown alga* Stypocaulon scoparium*) with solvents: water, water/methanol (1/1), methanol, and ethanol. Then the extracts were examined for the total phenolic content (TPC) using the Folin-Ciocalteu method. The highest amount of polyphenols was obtained for water extract (329 mg/100 g d.w. (dry weight)) and the lowest for ethanol extract (2.36 mg/100 g d.w.). Results showed a significant association between the antioxidant properties and TPC. The aqueous extract demonstrated the highest antioxidant activity and highest phenolic content. These results overlap with those reported by other researchers. Tierney et al. [42] observed that water used as the solvent for the extraction of phenolic compounds from Irish macroalgae (*Ascophyllum nodosum*,* Pelvetia canaliculata*,* Fucus spiralis,* and* Ulva intestinalis*) resulted in the highest extraction yields when compared with other solvents, for example, acetone/water (80 : 20) and ethanol/water (80 : 20). This reflects the hydrophilic nature of the majority of components found within macroalgal cells [42].

#### 3.1.3. Antibacterial Properties of Algal Extracts

Seaweeds contain large amounts of structurally diverse secondary metabolites which offer defense against pathogens, herbivores, and decaying organisms. Compounds that exhibit the bactericidal or bacteriostatic properties include amino acids, terpenoids, phlorotannins, acrylic acid, steroids, halogenated ketones and alkanes, cyclic polysulphides, fatty acids [43], proteins, polyphenols, polysaccharides, and pigments (e.g., chlorophyll and carotenoids) [22].

We examined the antibacterial activity of the extracts against gram-negative (*Escherichia coli*) and gram-positive (*Staphylococcus aureus*) bacteria. ES showed no inhibitory activities, whereas EB revealed an inhibitory activity (18 mm) against* E. coli* but did not have the zone of inhibition against* S. aureus*. The control group (gentamicin) showed the inhibitory zone (32 mm) against* E. coli *and* S. aureus* (26 mm). The literature data regarding the antibacterial properties of aqueous algal extracts are divergent. Mohana Priya and Ali [44] presented that the aqueous extract of* Ulva fasciata *showed the antibacterial activity against* E. coli* (16 mm) and* S. aureus* (15 mm). Christobel et al. [45] presented that 100%* Ulva fasciata* aqueous extract had inhibitory activity against* S. aureus* (10 mm) and* E. coli* (9 mm). Mansuya et al. [46] reported that aqueous extracts of* Ulva reticulata* did not inhibit* E. coli* growth, but* Cladophora glomerata* and* Ulva lactuca* extracts showed inhibition. Alghazeer et al. [47] wrote that aqueous extracts of* Ulva lactuca, Enteromorpha compressa, Enteromorpha prolifera,* and* Enteromorpha* spp. demonstrated the antimicrobial activity against* S. aureus* (11-12 mm) and* E. coli* (11-12 mm). Selvi et al. [48] observed that aqueous extracts of* Enteromorpha compressa, E*.* intestinalis, Ulva lactuca, *and* U*.* fasciata* showed trace antibacterial activity for both strains:* S. aureus* and* E. coli*. The differences in antibacterial properties of algal extracts may be caused by the composition of seaweeds, place, season of their collection, extraction techniques used, solvents (its polarity), and parameters of the extraction process [22, 49].

### 3.2. Utilitarian Properties of Algal Extracts

Seaweeds, due to their content of organic matter and fertilizer nutrients, have been used as soil conditioners for centuries [18]. The first practical method for liquefying seaweed for agricultural use was developed in 1949 [50]. Seaweed extract can produce beneficial effect on plants, such as early seed germination, improved crop yield, elevated resistance to abiotic and biotic stress, and also enhanced postharvest shelf-life of perishable products [18]. Great advantages of seaweed products, compared with conventional crop protection products, are that they are biodegradable, do not show toxicity, and exhibit activity in low doses (<15 L/ha) [50, 51]. In the present study we investigated the effect of algal extracts on total height, dry weight, content of chlorophyll and nutrients, and morphology of garden cress (*Lepidium sativum*). For the germination experiments, we prepared dilutions (0.5, 2.5, and 10%) of the raw extract.

#### 3.2.1. Total Height of the Cultivated Garden Cress

For both extracts, plant height (*N* = 20 from each group, from each replicate) was determined for all three dilutions (0.5, 2.5, and 10%). The extracts under study exhibited varying degrees of stimulatory effect on plant growth.  Table 2 presents the results. In most cases, plants in the experimental groups were higher than those in the control group, treated with distilled water. The best results were achieved for the plants treated with 10% EB; these were 13% longer than those in the control group. The lowest plants were observed in groups treated with 0.5% EB (by ~3.0% shorter than plants in the control group) and 2.5% EB (by ~5.0% shorter than control). The differences were statistically significant (for *p* < 0.05). Latique et al. [52] demonstrated that the application of extracts (25 and 50%), resulting from boiling the fresh biomass (*Ulva rigida*) in a distilled water, provided significant effects on bean growth (*Phaseolus vulgaris* L.). The best effect was noted in the group treated with a 25% dilution. Similar results were reported by Gireesh et al. [53], who tested extracts produced by boiling green alga* Ulva lactuca* in distilled water for an hour. Subsequently, the series of 5, 10, 20, 30, 40, and 50% concentrations were prepared and tested on seedling growth of* Vigna unguiculata* L. Walp, shoot and root length. The best results were obtained for the 20% aqueous seaweed extract. Higher concentrations (≥40%) yielded inhibited germination [53]. Kavipriya et al. [54] investigated the effect of extracts from different seaweeds including* Ulva lactuca*, produced by autoclaving the biomass with distilled water (121°C, 30 min), on* Vigna radiata* (green gram) seed germination and growth parameters. Seaweed liquid extracts were prepared with 0.1, 0.2, 0.3, 0.4, and 0.5% doses. The best results were obtained for 0.2% dilution: plants were 1.8 times higher than in the control. Pise and Sabale [32] investigated the effect of three seaweeds including* U. fasciata*. Different methods, using algal powder, were used for the preparation of extracts. In one of them, dried biomass was boiled for an hour in distilled water. In another, algae were soaked in distilled water for two days. The efficiency of the preparations (10, 25, and 50%) was examined on* Trigonella foenum-graecum* L. Shoot growth was increased by all the extracts and the maximum value was recorded for the 50% concentration.

#### 3.2.2. Weight of the Cultivated Plants

We found out that dry mass of the cultivated garden cress, taking into account both methods of the extraction and the dilutions of the extracts, was comparable in all the groups (Table 3). We observed no influence of algal extract concentration on* Lepidium sativum* dry weight. Gireesh et al. [53] showed that the 20% concentration of* Ulva lactuca* aqueous extract increased* Vigna unguiculata* L. Walp dry weight (plants were ~9% higher than in the control group). It was observed that higher concentrations (30, 40, and 50%) reduced the dry weight of plants. Kavipriya et al. [54] showed that the best results of extract (range of concentrations 0.1–0.5%) obtained from* Ulva lactuca*, in promoting the dry weight of plants, were in the group treated with 0.2% concentration (24% higher than in control group). In the Pise and Sabale [32] study, dry weight of plants was the highest when 50% concentration of* Ulva* extracts obtained by boiling and soaking methods was applied.

#### 3.2.3. Multielemental Composition of the Cultivated Garden Cress

The application of seaweed extracts can increase the content of micro- and macroelements in the cultivated plants [25, 55]. In the present study we observed that the highest content of micro- and macroelements in* Lepidium sativum* occurred in the groups treated with 0.5% EB, as well as 0.5% and 10% ES (Table 4). Among these three extracts, the best results were obtained for 10% ES. The content of B (76%), Cu (2.6 times), Mn (20%), Mo (48%), Ni (2.4 times), Zn (31%), K (15%), Mg (7%), Na (59%), and S (4%) was higher than in the control group. Michalak and Chojnacka [55] presented the results that showed that the cultivated garden cress contained mainly these micro- and macroelements, which occurred at the largest concentrations in the algal extract. Plants treated with 100% extract were rich in macroelements such as K (3 times more as compared to the control group treated with water), S (44% more), and Ca (about 35% more). Among the trace elements, the largest quantities in biomass were observed for B (5% more than in control) and Mn (2 times more).

#### 3.2.4. Chlorophyll Content in the Cultivated Cress

The concentrations of chlorophyll in cultivated plants are presented in Table 5. In most cases, total chlorophyll concentration in* Lepidium sativum* in experimental groups was higher than in the control group. The highest content of total chlorophyll in plant was in the group treated with 0.5% ES (2.5 time more than in the control group). It can be seen that, with increasing concentration of ES, the content of chlorophyll in plants decreased. For EB, the best results were obtained for the 2.5% concentration. A high content of elements such as Mg, Fe, and Cu in Baltic algae and consequently in extracts [25] and also the presence of betaine, which causes the increase of the concentration of chlorophyll in leaves [56], are related to the stimulatory effect on chlorophyll synthesis. Results of this study showed that the examined algal extracts increased plant productivity, resulting in increased chlorophyll content. Gireesh et al. [53] reported that lower concentrations of the aqueous* Ulva lactuca* extract have promoted the chlorophyll content of* Vigna unguiculata* (even about 20%). It was also noticed that the higher concentrations (>20%) decreased the chlorophyll content in plants. Gaikwad et al. [57] observed that foliar application of 0.1% aqueous extract of* Ulva lactuca* L. enhanced chlorophyll content in* Solanum melongena* L. when compared with control group. Osman and Salem [58] showed that the aquatic extracts (0.4 and 0.6%) obtained from* Ulva lactuca* significantly increased the content of chlorophyll* a* and* b *in sunflowers (*Helianthus annuus* L.). Pise and Sabale [32] observed that extracts obtained from* Ulva* increased the content of photosynthetic pigments in harvested* Trigonella foenum-graecum* L.

#### 3.2.5. SEM Analysis of Cultivated Plants

**Figure 1:**
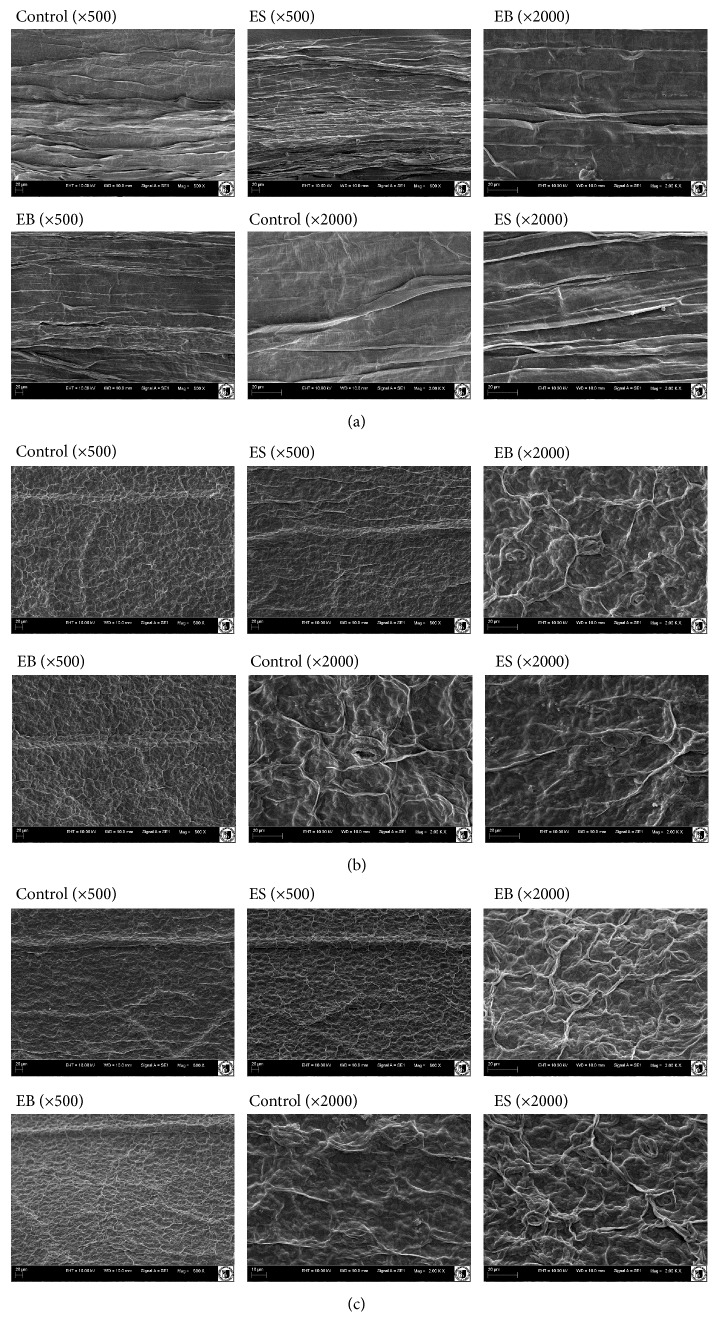
The influence of algal extracts produced by boiling and soaking methods on plant morphology: (a) stalk, (b) leaf, internal part, and (c) leaf, external part of plant.

In the present paper, to evaluate the effect of EB and ES extracts on* Lepidium sativum* morphology, stalk, and leaf (the internal and external part) we used Scanning Electron Microscopy.  Figure 1 presents observations for two magnifications (500 and 2000). The morphological studies showed significant stem changes treated with both aqueous extracts. In the group treated with EB, we observed the skin with clearly setting parallel fibers of the surface layer. In the case of garden cress treated with ES it can be noticed that the surface layer of the stalk (Figure 1(a)) was significantly shrunken. SEM showed also a considerable morphological changes of the lower epidermis of the plant leaves treated with both extracts. In both groups we observed the shrinking of the cuticles and the stoma (Figure 1(b)). This study showed slight changes in an external part of epidermal leaf and the enlargement of the stomas (Figure 1(c)). Morphological studies showed the impacts of the extracts on garden cress morphology, mainly in stoma composition and size.

## 4. Conclusions

Considering the above findings, the seaweed extracts derived from* Polysiphonia, Ulva*, and* Cladophora* could be used as nontoxic biostimulants of plant growth. It was observed that obtained extracts were similar in terms of the concentration of the microelements and macroelements and low content of toxic elements. This research shows that water is a good solvent to extract phenolic compounds. In most cases, algal extracts did not show antibacterial activity against* E. coli* and* S. aureus*. Only the EB extract presented an inhibitory activity against* E. coli*. Germination tests showed a positive influence of obtained products on height, multielemental composition, and the content of chlorophyll in the cultivated plants (*Lepidium sativum*) and also showed the impacts on morphology of garden cress. The best product which increased the total height of garden cress was 10% EB. Plants in this group were 13% longer than in the control group. The extract which decreased plant length by 3% was 0.5% EB. The dry mass of the cultivated garden cress was comparable in all groups. Mainly 10% ES influenced content of micro- and macroelements in* Lepidium sativum*. In the group treated with 0.5% ES we observed the highest content of total chlorophyll in plant. Used seaweed species can be considered as a potential source of nutrients for plants and be used in agriculture and horticulture to attain better germination, growth, and yield. Because of the reported multifunctional properties of seaweed extracts, their exploitation as a source of biological active compounds could be possible.

## Figures and Tables

**Table 1:** Multielemental composition of raw algal biomass and extracts obtained by boiling and soaking in water.

Element	Baltic seaweeds (mg/kg d.m.) (*N* = 3)	EB (*N* = 3)	ES (*N* = 3)	Extract obtained by boiling (45 min, 60°C) *Ulva reticulata*	Extract obtained by boiling *Ulva lactuca*	Extract obtained by MAE 25°C	Extract obtained by MAE 40°C	Extract obtained by MAE 60°C
				[34]	[35]	[25]		
	mg/L							
Macroelements								
Ca	40292 ± 8058	333 ± 50	410 ± 61	158	—	354 ± 53	363 ± 54	365 ± 54
K	5082 ± 1016	969 ± 145	978 ± 147	175	0.980	868 ± 130	901 ± 135	951 ± 142
Mg	3181 ± 636	300 ± 45	357 ± 54	108	9.28	303 ± 45	311 ± 46	322 ± 48
Na	6354 ± 1271	1239 ± 248	1302 ± 260	295	5.04	1050 ± 211	1200 ± 240	1250 ± 250
P	1155 ± 231	34.7 ± 5.2	5.22 ± 0.78	—	—	9.52 ± 1.43	18.3 ± 2.7	32.9 ± 4.9
S	8614 ± 1723	670 ± 100	599 ± 90	—	—	562 ± 84	582 ± 87	702 ± 105

Microelements								
B	97.8 ± 14.7	6.50 ± 0.97	2.62 ± 0.39	—	—	3.30 ± 0.5	3.44 ± 0.51	4.74 ± 0.71
Co	2.84 ± 0.43	0.0100 ± 0.0025	0.0100 ± 0.0025	—	0.201	0.012 ± 0.003	0.012 ± 0.003	0.0135 ± 0.0034
Cu	12.7 ± 1.9	0.140 ± 0.021	0.0200 ± 0.005	1.15	3.02	0.117 ± 0.0117	0.148 ± 0.022	0.108 ± 0.016
Fe	6660 ± 1332	2.53 ± 0.38	17.6 ± 2.6	5.22	0.417	1.19 ± 0.2	2.17 ± 0.3	4.47 ± 0.70
Mn	232 ± 35	2.43 ± 0.36	3.71 ± 0.56	—	0.050	2.52 ± 0.37	2.62 ± 0.39	3.07 ± 0.46
Mo	0.310 ± 0.046	0.0200 ± 0.005	0.000	—	—	0.001 ± 0.000	0.179 ± 0.026	0.0108 ± 0.0027
Ni	5.23 ± 0.78	0.130 ± 0.019	0.120 ± 0.018	—	—	0.108 ± 0.016	0.111 ± 0.016	0.132 ± 0.019
Si	907 ± 136	9.10 ± 1.36	9.73 ± 1.46	—	—	3.10 ± 0.46	6.69 ± 1	11.9 ± 1.8
Zn	64.9 ± 9.7	0.240 ± 0.036	0.100 ± 0.015	1.25	1.02	0.0746 ± 0.0112	0.201 ± 0.03	0.169 ± 0.025

Toxic metals								
As	3.90 ± 0.507	0.170 ± 0.022	0.160 ± 0.021	—	—	0.246 ± 0.032	0.150 ± 0.019	0.198 ± 0.025
Cd	0.710 ± 0.092	<LLD	<LLD	—	—	<LLD	0.001 ± 0.000	0.001 ± 0.000
Pb	7.03 ± 0.91	0.0400 ± 0.008	0.0100 ± 0.0025	—	—	0.0098 ± 0.002	0.0104 ± 0.0021	0.032 ± 0.006

<LLD: below limit of detection.

**Table 2:** The results of total height of the cultivated garden cress in the examined groups (*N* = 3).

Group	Average height of cress (cm) ± SD^*∗*^
C	5.36 ± 0.59^a^
EB 0.5%	5.21 ± 0.59^bcde^
EB 2.5%	5.10 ± 0.71^fghi^
EB 10%	6.07 ± 0.61^abf^
ES 0.5%	5.93 ± 0.75^cg^
ES 2.5%	5.78 ± 0.93^dh^
ES 10%	5.90 ± 0.86^ei^

^*∗*^Three replicates for each group; 20 randomly selected plants in each replicate were measured.

a, b, c, d,…: statistically significant differences for *p* < 0.05.

**Table 3:** The dry weight of garden cress in various experimental groups (*N* = 3).

Group	Average dry weight of cress (g) ± SD^*∗*^
C	0.0739 ± 0.0033
EB 0.5%	0.0727 ± 0.0037
EB 2.5%	0.0670 ± 0.0016
EB 10%	0.0733 ± 0.0033
ES 0.5%	0.0712 ± 0.0022
ES 2.5%	0.0680 ± 0.0010
ES 10%	0.0703 ± 0.0014

^*∗*^Three replicates for each group; all plants in each replicate were weighed.

**Table 4:** Multielemental composition of the cultivated garden cress (mg/kg d.m., *N* = 3).

Element	C	EB			ES		
		0.5%	2.5%	10%	0.5%	2.5%	10%
Macroelements							
Ca	11161 ± 394	12752 ± 279	13126 ± 693	12956 ± 902	10818 ± 508	11881 ± 288	10475 ± 644
K	58067 ± 3802	65020 ± 291	67021 ± 1547	67774 ± 1430	68562 ± 190	67774 ± 2705	66983 ± 623
Mg	6974 ± 68	7188 ± 419	7210 ± 2	6854 ± 340	7662 ± 307	7409 ± 35	7456 ± 53
Na	1850 ± 8	1848 ± 181	2140 ± 24	3132.50 ± 92.79	2152.53 ± 97.27	2236.00 ± 170.10	2933.15 ± 51.10
P	16475 ± 964	16626 ± 798	16236 ± 301	14727 ± 686	16390 ± 170	17197 ± 211	16550 ± 83
S	15508 ± 939	15139 ± 488	14921 ± 166	12786 ± 999	15193 ± 260	17572 ± 1009	16155 ± 236

Microelements							
B	7.30 ± 0.82	11.3 ± 0.2	12.7 ± 0.52	12.7 ± 3.1	20.8 ± 13.0	15.1 ± 3.0	12.8 ± 0.9
Co	0.08 ± 0.03	0.11	0.09 ± 0.03	0.04 ± 0.01	0.12 ± 0.12	0.12 ± 0.05	0.21 ± 0.13
Cu	4.24 ± 0.07	5.75 ± 0.65	5.43 ± 0.85	4.72 ± 0.04	6.25 ± 1.21	9.80 ± 5.46	11.19 ± 2.49
Fe	278 ± 11	288 ± 46	279 ± 33	263 ± 13	198.79 ± 6.07	207 ± 3.0	227.77 ± 0.96
Mn	41.9 ± 1.85	47.6 ± 3.2	46.1 ± 1.6	45.0 ± 2.3	49.09 ± 0.13	49.04 ± 0.62	50.39 ± 1.00
Mo	1.20 ± 0.01	2.17 ± 0.47	1.73 ± 0.07	1.89 ± 0.14	1.79 ± 0.16	1.63 ± 0.14	1.77 ± 0.09
Ni	0.63 ± 0.16	1.44 ± 0.54	1.10 ± 0.02	0.84 ± 0.23	1.26 ± 0.13	1.23 ± 0.63	1.51 ± 0.13
Si	182 ± 22	271 ± 35	244 ± 38	226 ± 8.0	245 ± 71.49	192.25 ± 20.15	180.45 ± 8.41
Zn	58.22 ± 0.71	81.9 ± 2.7	88.2 ± 4.1	76.8 ± 0.2	88.6 ± 2.18	83.16 ± 0.04	76.35 ± 0.24

Toxic metals							
As	0.35 ± 0.12	0.17 ± 0.07	0.0521 ± 0.0104	0.36 ± 0.05	0.49 ± 0.59	0.15 ± 0.10	<LLD
Cd	0.26 ± 0.04	0.28 ± 0.01	0.24 ± 0.01	0.21 ± 0.01	0.31 ± 0.02	0.30 ± 0.02	0.24 ± 0.03
Pb	1.19 ± 0.01	1.52 ± 0.39	1.48 ± 0.05	1.53 ± 0.46	1.92 ± 0.62	1.44 ± 0.09	1.28 ± 0.01

**Table 5:** Chlorophyll concentration in the cultivated garden cress (mg/L).

Sample	Concentration of chlorophyll *a*	Concentration of chlorophyll *b*	Total chlorophyll concentration
C	15.5	5.95	21.4
EB 0.5%	23.9	9.00	32.9
EB 2.5%	26.4	9.55	36.0
EB 10%	14.9	5.97	20.8
ES 0.5%	12.2	42.2	54.4
ES 2.5%	10.0	34.9	44.9
ES 10%	9.4	32.7	42.1

## References

[1] Sridhar S., Rengasamy R. (2011). Influence of seaweed liquid fertilizer on growth and biochemical characteristics of *Arachis hypogea* L. under field trial. *Journal of Ecobiotechnology*.

[2] Wijesinghe W. A. J. P., Jeon Y.-J. (2012). Enzyme-assistant extraction (EAE) of bioactive components: a useful approach for recovery of industrially important metabolites from seaweeds: a review. *Fitoterapia*.

[3] Athukorala Y., Lee K.-W., Song C. (2003). Potential antioxidant activity of marine red alga *Grateloupia filicina* extracts. *Journal of Food Lipids*.

[4] Thomas N. V., Kim S.-K. (2013). Beneficial effects of marine algal compounds in cosmeceuticals. *Marine Drugs*.

[5] Michalak I., Chojnacka K. (2015). Algae as production systems of bioactive compounds. *Engineering in Life Sciences*.

[6] Chojnacka K., Saeid A., Witkowska Z., Tuhy Ł. (2012). Biologically active compounds in seaweed extracts—the prospects for the application. *The Open Conference Proceedings Journal*.

[7] Yuan Y. V., Walsh N. A. (2006). Antioxidant and antiproliferative activities of extracts from a variety of edible seaweeds. *Food and Chemical Toxicology*.

[8] Nwosu F., Morris J., Lund V. A., Stewart D., Ross H. A., McDougall G. J. (2011). Anti-proliferative and potential anti-diabetic effects of phenolic-rich extracts from edible marine algae. *Food Chemistry*.

[9] Ye H., Wang K., Zhou C., Liu J., Zeng X. (2008). Purification, antitumor and antioxidant activities *in vitro* of polysaccharides from the brown seaweed *Sargassum pallidum*. *Food Chemistry*.

[10] Khan M. N. A., Choi J. S., Lee M. C. (2008). Anti-inflammatory activities of methanol extracts from various seaweed species. *Journal of Environmental Biology*.

[11] Samee H., Li Z.-X., Lin H., Khalid J., Guo Y.-C. (2009). Anti-allergic effects of ethanol extracts from brown seaweeds. *Journal of Zhejiang University: Science B*.

[12] Schaeffer D. J., Krylov V. S. (2000). Anti-HIV activity of extracts and compounds from algae and cyanobacteria. *Ecotoxicology and Environmental Safety*.

[13] Holdt S. L., Kraan S. (2011). Bioactive compounds in seaweed: functional food applications and legislation. *Journal of Applied Phycology*.

[14] Lordan S., Ross R. P., Stanton C. (2011). Marine bioactives as functional food ingredients: potential to reduce the incidence of chronic diseases. *Marine Drugs*.

[15] Sivasankari S., Venkatesalu V., Anantharaj M., Chandrasekaran M. (2006). Effect of seaweed extracts on the growth and biochemical constituents of *Vigna sinensis*. *Bioresource Technology*.

[16] Michalak I., Chojnacka K. (2008). The application of macroalga *Pithophora varia* Wille enriched with microelements by biosorption as biological feed supplement for livestock. *Journal of the Science of Food and Agriculture*.

[17] Fleurence J. (1999). Seaweed proteins: biochemical, nutritional aspects and potential uses. *Trends in Food Science and Technology*.

[18] Khan W., Rayirath U. P., Subramanian S. (2009). Seaweed extracts as biostimulants of plant growth and development. *Journal of Plant Growth Regulation*.

[19] Rathore S. S., Chaudhary D. R., Boricha G. N. (2009). Effect of seaweed extract on the growth, yield and nutrient uptake of soybean (*Glycine max*) under rainfed conditions. *South African Journal of Botany*.

[20] Gupta V., Kumar M., Brahmbhatt H., Reddy C. R. K., Seth A., Jha B. (2011). Simultaneous determination of different endogenetic plant growth regulators in common green seaweeds using dispersive liquid-liquid microextraction method. *Plant Physiology and Biochemistry*.

[21] Chojnacka K. (2012). Algal extracts. Biological concentrate of the future. *Przemysl Chemiczny*.

[22] Michalak I., Chojnacka K. (2014). Algal extracts: technology and advances. *Engineering in Life Sciences*.

[23] Yang B., Jiang Y., Shi J., Chen F., Ashraf M. (2011). Extraction and pharmacological properties of bioactive compounds from longan (*Dimocarpus longan* Lour.) fruit- a review. *Food Research International*.

[24] Rodriguez-Jasso R. M., Mussatto S. I., Pastrana L., Aguilar C. N., Teixeira J. A. (2011). Microwave-assisted extraction of sulfated polysaccharides (fucoidan) from brown seaweed. *Carbohydrate Polymers*.

[25] Michalak I., Tuhy Ł., Chojnacka K. (2015). Seaweed extract by microwave assisted extraction as plant growth biostimulant. *Open Chemistry*.

[26] Cheung P. C. K., Leung A. Y. H., Ang P. O. (1998). Comparison of supercritical carbon dioxide and soxhlet extraction of lipids from a brown seaweed, *Sargassum hemiphyllum* (Turn.) C. Ag. *Journal of Agricultural and Food Chemistry*.

[27] Billakanti J. M., Catchpole O. J., Fenton T. A., Mitchell K. A., Mackenzie A. D. (2013). Enzyme-assisted extraction of fucoxanthin and lipids containing polyunsaturated fatty acids from *Undaria pinnatifida* using dimethyl ether and ethanol. *Process Biochemistry*.

[28] Zhu X.-J., An X.-X., Gu L., Hu Q.-H. (2008). Optimization technique of synchronous ultrasonic-assisted extraction of polysaccharide and phycobiliprotein from *Porphyra yezoensis*. *Food Science*.

[29] Yi Z., Yin-Shan C., Hai-Sheng L. (2001). Screening for antibacterial and antifungal activities in some marine algae from the Fujian coast of China with three different solvents. *Chinese Journal of Oceanology and Limnology*.

[30] Ganesan P., Kumar C. S., Bhaskar N. (2008). Antioxidant properties of methanol extract and its solvent fractions obtained from selected Indian red seaweeds. *Bioresource Technology*.

[31] Wilk R., Chojnacka K., Rój E., Górecki H. (2014). Technology for preparation of algae extract. Part 1. Raw material. *Przemysł Chemiczny*.

[32] Pise N. M., Sabale A. B. (2010). Effect of seaweed concentrates on the growth and biochemical constituents of *Trigonella Foenum-Graecum* L.. *Journal of Phytology*.

[33] Arnon D. I. (1949). Copper enzymes in isolated chloroplasts. Polyphenoloxidase in *Beta vulgaris*. *Plant Physiology*.

[34] Selvam G. G., Sivakumar K. (2013). Effect of foliar spray from seaweed liquid fertilizer of *Ulva reticulata* (Forsk.) on *Vigna mungo* L. and their elemental composition using SEM-energy dispersive spectroscopic analysis. *Asian Pacific Journal of Reproduction*.

[35] Sivasangari R. S., Nagaraj S., Vijayanand N. (2010). Biofertilizing efficiency of brown and green algae on growth, biochemical and yield parameters of *Cyamopsis tetragonal laba* (L.) Taub. *Recent Research in Science and Technology*.

[36] Burtin P. (2003). Nutritional value of sea seaweeds. *Electronic Journal of Environmental, Agricultural and Food Chemistry*.

[37] Wang T., Jónsdóttir R., Ólafsdóttir G. (2009). Total phenolic compounds, radical scavenging and metal chelation of extracts from Icelandic seaweeds. *Food Chemistry*.

[38] Cox S., Abu-Ghannam N., Gupta S. (2010). An assessment of the antioxidant and antimicrobial activity of six species of edible Irish seaweeds. *International Food Research Journal*.

[39] Zhang Q., Zhang J., Shen J., Silva A., Dennis D. A., Barrow C. J. (2006). A simple 96-well microplate method for estimation of total polyphenol content in seaweeds. *Journal of Applied Phycology*.

[40] Devi K. P., Suganthy N., Kesika P., Pandian S. K. (2008). Bioprotective properties of seaweeds: *in vitro* evaluation of antioxidant activity and antimicrobial activity against food borne bacteria in relation to polyphenolic content. *BMC Complementary and Alternative Medicine*.

[41] López A., Rico M., Rivero A., Suárez de Tangil M. (2011). The effects of solvents on the phenolic contents and antioxidant activity of *Stypocaulon scoparium* algae extracts. *Food Chemistry*.

[42] Tierney M. S., Smyth T. J., Hayes M., Soler-Vila A., Croft A. K., Brunton N. (2013). Influence of pressurised liquid extraction and solid-liquid extraction methods on the phenolic content and antioxidant activities of Irish macroalgae. *International Journal of Food Science and Technology*.

[43] Shanmughapriya S., Manilal A., Sujith S., Selvin J., Kiran G. S., Natarajaseenivasan K. (2008). Antimicrobial activity of seaweeds extracts against multiresistant pathogens. *Annals of Microbiology*.

[44] Mohana Priya K., Ali S. K. (2011). Antibacterial activity of aqueous extract of sea weed Ulva fasciata: an in vitro study. *International Journal of Current Pharmaceutical Review and Research*.

[45] Christobel J. G., Lipton A. P., Aishwarya M. S., Sarika A. R., Udayakumar A. (2011). Antibacterial activity of aqueous extract from selected macroalgae of southwest coast of India. *Seaweed Research Utilization*.

[46] Mansuya P., Aruna P., Sridhar S., Kumar J. S., Babu S. (2010). Antibacterial activity and qualitative phytochemical analysis of selected seaweeds from Gulf of Mannar region. *Journal of Experimental Sciences*.

[47] Alghazeer R., Whida F., Abduelrhman E., Gammoudi F., Azwai S. (2013). Screening of antibacterial activity in marine green, red and brown macroalgae from the western coast of Libya. *Natural Science*.

[48] Selvi M., Selvaraj R., Chidambaram A. (2001). Screening for antibacterial activity of macro algae. *Seaweed Research and Utilization*.

[49] Varier K. M., Milton M. C. J., Arulvasu C., Gajendran B. (2013). Evaluation of antibacterial properties of selected red seaweeds from Rameshwaram, Tamil Nadu, India. *Journal of Academia and Industrial Research*.

[50] Craigie J. S. (2011). Seaweed extract stimuli in plant science and agriculture. *Journal of Applied Phycology*.

[51] Tuhy Ł., Witkowska Z., Saeid A., Chojnacka K. (2012). Use of seaweed extracts for production of fertilizers, feed, food and cosmetics. *Przemysł Chemiczny*.

[52] Latique S., Chernane H., Mansori M., El Kaoua M. (2013). Seaweed liquid fertilizer effect on physiological and biochemical parameters of bean plant (*Phaesolus vulgaris*variety *Paulista*) under hydroponic system. *European Scientific Journal*.

[53] Gireesh R., Haridevi C. K., Salikutty J. (2011). Effect of *Ulva lactuca* extract on growth and proximate composition of *Vigna unguiculata* l. Walp. *Journal of Research in Biology*.

[54] Kavipriya R., Dhanalakshmi P. K., Jayashree S., Thangaraju N. (2011). Seaweed extract as a biostimulant for legume crop, green gram. *Journal of Ecobiotechnology*.

[55] Michalak I., Chojnacka K. (2013). Use of extract from Baltic seaweeds produced by chemical hydrolysis in plant cultivation. *Przemysł Chemiczny*.

[56] Whapham C. A., Blunden G., Jenkins T., Hankins S. D. (1993). Significance of betaines in the increased chlorophyll content of plants treated with seaweed extract. *Journal of Applied Phycology*.

[57] Gaikwad S., Pingle S. D., Khose R. G. (2012). Effect of foliar spray of *Ulva lactic* L. on chemical contents and morphological parameters of *Solanum melongena* L. VAR. ‘Pancha Ganga’. *Bionano Frontier*.

[58] Osman H. E., Salem O. M. A. (2011). Effect of seaweed extracts as foliar spray on sunflower yield and oil content. *Egyptian Journal of Phycology*.

